# Reply to ‘Implantable cardioverter-defibrillators might not be necessary in all patients with idiopathic ventricular fibrillation’

**DOI:** 10.1007/s12471-024-01899-z

**Published:** 2024-09-09

**Authors:** Lisa M. Verheul, Rutger J. Hassink

**Affiliations:** Department of Cardiology, UMC Utrecht, Utrecht, The Netherlands

Dear Editor,

We thank Dr. Noordman and Dr. Maass for their interest in our study and for highlighting the interesting topic regarding the need for (replacement of) an implantable cardioverter defibrillator (ICD) in idiopathic ventricular fibrillation (iVF) patients [1].

We found similar results as mentioned by the authors regarding appropriate ICD therapy [2, 3]. A major goal of our registry is to identify patients at risk for recurrences by discovering the substrate and developing targeted therapy. Thereby preventing inappropriate ICD therapy. However, we have not been able to achieve this goal, leading to a persistent ICD indication for all iVF patients according to the ESC guideline.

Our median follow-up period (6 [2–12] years) is too short to provide general recommendations on replacing empty ICD’s. However, in specific cases, e.g. when complications challenge re-implantation, not replacing the ICD may indeed be considered, with the pros and the cons weighed in a shared decision making setting. In the future we hope to provide more guidance by extending follow-up of our patients.
